# Protein Supramolecular Structures: From Self-Assembly to Nanovaccine Design

**DOI:** 10.3390/nano10051008

**Published:** 2020-05-25

**Authors:** Ximena Zottig, Mélanie Côté-Cyr, Dominic Arpin, Denis Archambault, Steve Bourgault

**Affiliations:** 1 Department of Chemistry, Université du Québec à Montréal, Montreal, QC H2L 2C4, Canada; zottig.ximena_alumine@courrier.uqam.ca (X.Z.); cote-cyr.melanie@courrier.uqam.ca (M.C.-C.); arpin.dominic@courrier.uqam.ca (D.A.); 2 The Quebec Network for Research on Protein Function, Engineering and Applications, PROTEO, Quebec, QC G1V 0A6, Canada; 3 The Swine and Poultry Infectious Diseases Research Centre, CRIPA, Saint-Hyacinthe, QC J2S 2M2, Canada; 4 Department of Biological Sciences, Université du Québec à Montréal, Montreal, QC H2L 2C4, Canada

**Keywords:** self-assembly, vaccine, supramolecular structure, nanovaccine, nanoassemblies, immune response, adjuvant, protein

## Abstract

Life-inspired protein supramolecular assemblies have recently attracted considerable attention for the development of next-generation vaccines to fight against infectious diseases, as well as autoimmune diseases and cancer. Protein self-assembly enables atomic scale precision over the final architecture, with a remarkable diversity of structures and functionalities. Self-assembling protein nanovaccines are associated with numerous advantages, including biocompatibility, stability, molecular specificity and multivalency. Owing to their nanoscale size, proteinaceous nature, symmetrical organization and repetitive antigen display, protein assemblies closely mimic most invading pathogens, serving as danger signals for the immune system. Elucidating how the structural and physicochemical properties of the assemblies modulate the potency and the polarization of the immune responses is critical for bottom-up design of vaccines. In this context, this review briefly covers the fundamentals of supramolecular interactions involved in protein self-assembly and presents the strategies to design and functionalize these assemblies. Examples of advanced nanovaccines are presented, and properties of protein supramolecular structures enabling modulation of the immune responses are discussed. Combining the understanding of the self-assembly process at the molecular level with knowledge regarding the activation of the innate and adaptive immune responses will support the design of safe and effective nanovaccines.

## 1. Introduction

Vaccination has constituted the strategy of choice to prevent the propagation infectious diseases and has shown tremendous potential for treatment of cancer and autoimmune diseases [1,2]. Modern vaccine technology has shifted toward the usage of defined molecular components, such as microbial or cancer-specific antigens [3]. In comparison to inactivated or live-attenuated pathogen-based vaccines, subunit vaccines constitute safer formulations, although they tend to be weakly immunogenic and to require the use of non-specific immunostimulants [4]. Co-delivery of an antigen with adjuvant allows its stabilization, sustained release and/or specific activation of the immune system, potentiating the immune response. Whereas numerous immunostimulators were investigated, such as aluminum salts and saponin-based adjuvants, their mechanisms of action and biophysical properties remain challenging to study because of the structural heterogeneity of the particulates [5]. Pathogen-associated molecular patterns (PAMPs) and toll-like receptor (TLR) agonists are known to enhance innate and adaptive immune responses, although clinical usage of these ligands is limited by off-target inflammation and side effects [6]. An alternative strategy to increase the immunogenicity of subunit vaccines consists of developing nanoparticles exposing a high density of the relevant antigens. These nanovaccines combine numerous advantages, including improved immunogenicity, increased uptake by antigen-presenting cells (APCs) and stabilization of the antigen [7]. Synthetic and inorganic nanoparticles, such as polymers and gold nanoparticles, were evaluated to enhance antigen processing and/or as non-specific immunostimulants [8]. However, these nanoparticles present limitations associated with their toxicity, low stability and/or poor biocompatibility. Subunit nanovaccines composed of proteins that self-assemble into well-organized repetitive antigen display platforms have recently attracted interest for their unique properties, such as biocompatibility, enhanced stability, molecular specificity and multivalency, leading to efficient delivery and presentation of antigenic determinants [9]. Moreover, harnessing the architecture of virus in terms of size, shape and symmetry, with defined proteinaceous suprastructures, may be the key for developing self-adjuvanted subunit vaccines. Over the last two decades, the interest of using self-assembling peptides and proteins to conceive supramolecular assemblies for nanovaccine design has increased considerably [7]. In this review, after briefly introducing the fundamentals of supramolecular interactions involved in protein self-assembly, we present the strategies to design and functionalize protein nanostructures. Finally, we highlight some of the recent and elegant works on nanovaccine design and discuss the structural and physicochemical properties of the protein assemblies that modulate the potency and the polarization of the immune responses.

## 2. Interactions Guiding Protein Self-Assembly

Protein self-assembly represents a unique strategy to generate organized nanoparticles with tailored function. However, the mechanisms guiding self-recognition and ordered aggregation of polypeptides into defined nanostructures are complex and remain, in many cases, poorly defined. Precise control over this bottom-up assembly process and over the final supramolecular architecture still constitutes a critical challenge for the design of protein-based nanoparticles [10]. The rational design of proteinaceous assemblies requires a deep understanding of the molecular basis governing self-assembly and molecular structure, including hydrogen bonds, as well as electrostatic, hydrophobic and π-π interactions [11,12]. Complex interplay between these biomolecular interactions results in defined self-assembling motifs, such as coiled-coil and cross-β-sheet, which support the rational bottom-up design of next-generation vaccine scaffold. In this section, we first present the intra- and intermolecular forces driving self-assembly and discuss how this knowledge can be integrated to engineer protein-based nanovaccines.

### 2.1. Fundamentals of Molecular Self-Assembly

Proteinaceous assemblies can be described by a delicate balance of non-covalent interactions that are dictated by amino acid side-chains and the polyamide peptide backbone [13]. The long range and relative weakness of non-covalent interactions are compensated by polyvalence and abundance of these interactions, allowing the formation of diverse mesoscopic structures [14].

Hydrophobic interactions: As hydrophobic interactions minimize the energy penalty associated with the exposition of a residue with a hydrophobic interface into water, the hydrophobic effect is key for protein self-assembly and has been harnessed to generate well-defined supramolecular structures [15,16]. For instance, vesicles, micelles and filaments are assemblies that mainly arise from hydrophobic interactions [17,18,19,20]. These assemblies are often composed of small amphiphilic peptide building blocks, which have a hydrophobic tail and hydrophilic head, an organization mimicking surfactants [21,22,23]. Peptide micelles, in which the building blocks are composed of aspartic acid residues (hydrophilic head) conjugated to repeats of valine or leucine residues (hydrophobic tail), have been evaluated as drug delivery systems [21,22].

Hydrogen bonding: While the hydrophobic effect provides the thermodynamic forces to protein folding and self-recognition, hydrogen bonding often confers directionality and specificity [24]. Accordingly, hydrogen bonds play an important role in protein self-assembly into nanoparticles, including the formation of coiled-coil and cross-β-sheet assembling motifs [25,26]. Self-assembly of the RADA16-I peptide into ordered β-sheet-based assemblies occurs through the formation of a network of hydrogen bonds [27,28]. Similarly, hydrogen bonds play a critical role in the lateral association and alignment of Q11-based nanofibrils, which has been evaluated as an immunostimulant [29,30].

Ionic bonding: Ionizable residue side-chains contribute to the higher hierarchical organization of protein nanostructures. Electrostatic interactions involving proteins are complex, since the microenvironment surrounding proteins is not homogeneous, implicating local effects between proximal charges and interatomic contacts [31]. Furthermore, the strong energy penalty associated with burying charged residues into the nonpolar environment of protein cores prevents aggregation and contributes to solubility. While electrostatic interactions can inhibit protein self-association, they can also guide self-assembly and control the final morphology. For instance, it has been recently shown that the morphology and homogeneity of amyloid-based nanoparticles can be controlled by the addition of charged capping units at the N-terminus of a self-assembling amyloidogenic peptide [32].

π-π and cation-π interactions: In protein-based assemblies, π-stacking refers to interactions occurring between neighboring aromatic rings (π-π) or between cations and neighboring aromatic rings (cation-π) [33,34]. For instance, the addition of aromatic residues (e.g., Tyr and Phe) at both ends of the triple-helical peptide Pro-Hyp-Gly, used as a model of collagen filaments, promotes ordered aggregation, leading to insoluble and well-defined linear fibrils [35,36]. Interestingly, cation-π interactions were exploited to tune the underwater adhesion of a peptide film, indicating that this non-covalent interaction is promising to design proteinaceous nanoparticles with adhesion properties [37].

### 2.2. The Interplay of Non-Covalent Interactions

**Figure 1:**
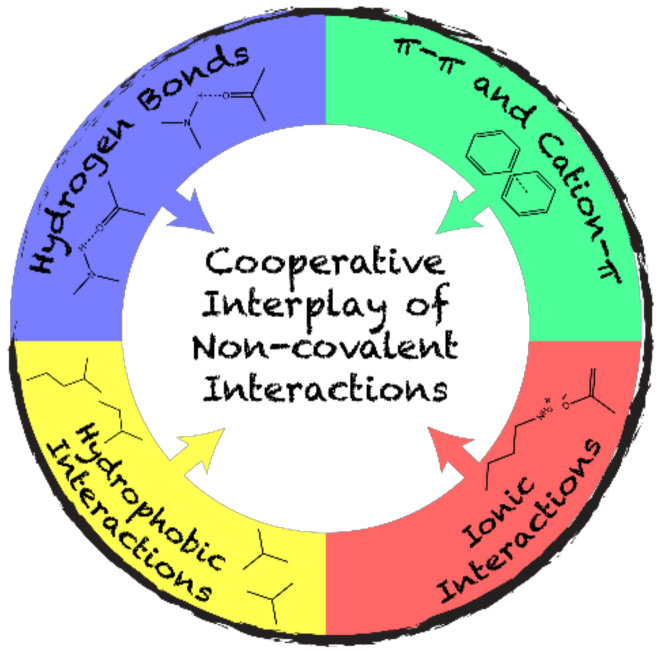
Schematic representation of the complex cooperative interplay of non-covalent interactions driving protein self-assembly.

Considering their complexity, biological supramolecular assemblies are the result of a fine spatiotemporal orchestration of non-covalent interactions (Figure 1) which compensates for the entropy costs of self-assembly processes [38,39]. Self-assembly is achieved through the combination of hydrophobic interactions, hydrogen and ionic bonding, and π-stacking. In this cooperative process, dominant interactions guide self-recognition, and the final architecture is usually under thermodynamic control. For example, cross-β fibril formation is usually ascribed to a nucleation-dependent self-assembly. In this process, hydrophobic interactions are the primary thermodynamic driving forces, while ionic bonding provides specificity in nucleation, and hydrogen bonding and π-π interactions stabilize intermolecular β-sheets [40,41]. The microenvironment plays a key role on the final architectures. By varying the external conditions of self-assembly, a dominant interaction may be overcome by another. This could lead to changes in the kinetics of self-assembly, and in the morphology and properties of the final supramolecular structures [42]. In response to environmental conditions, flagellin, which is currently evaluated in clinical trial as a vaccine nanoplatform, can adopt different mesoscopic morphologies, leading to diverse immunological responses [43,44]. In addition, the abovementioned interactions also contribute to the mucosal adhesion of particles, and this contribution is important for the intranasal delivery of nanovaccines [8].

## 3. Self-Assembling Motifs

Self-assembling motifs are the fundamental building elements of a wide range of advanced protein-based nanoparticles. Hence, assemblies can be engineered by precisely organizing oligomerization domains and self-assembling motifs. This requires deep knowledge and unique prediction of the complex interactions involved. Since precise de novo prediction tools are still limited, the most efficient strategies to design large protein supramolecular structures remain based on nature-derived self-assembling sequences and domains [45,46]. In this section, we present the most common life-inspired self-assembling motifs that support the engineering of versatile and ordered nanostructures for vaccine applications.

### 3.1. Coiled-Coil Motifs

**Figure 2:**
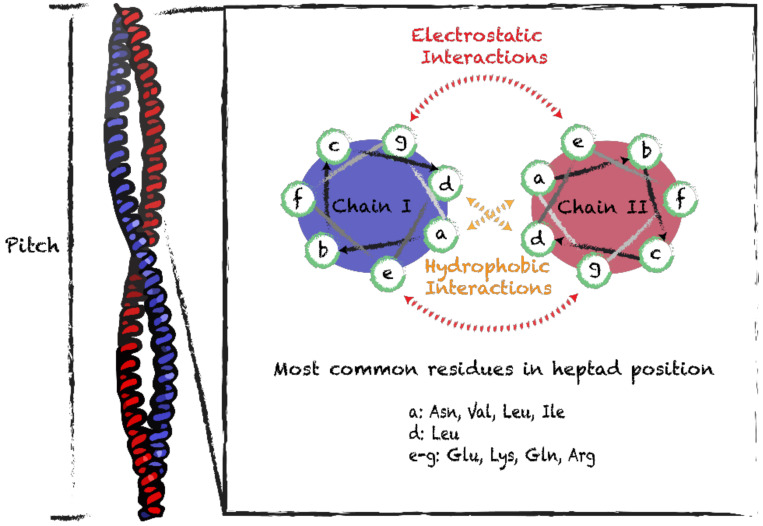
Schematic representation of the coiled-coil supramolecular structure and helical wheels of heptad sequences. Side view of the coiled-coil structure (PDB 1C1G) and characteristic helical pitch. Interactions within homodimers and most frequently observed residues in positions a, d, e and g of heptad sequences.

Because of their simplicity and regularity, coiled-coil motifs represent one of the most prevalent building blocks in protein nanoassemblies. These motifs consist of two or more α-helixes wrapping around each other through hydrophobic and ionic interactions. The canonical arrangement assumes a left-handed supercoiled structure with a regular packing that requires periodicity described by helical symmetry. This coiled-coil structure presents a seven-residue periodicity (3.5 residues per turn) with a pitch angle of 20° [47]. Ordered helical coiled-coil motifs can be engineered by combining in tandem 3 or 4 of heptad repeats (Figure 2) [48]. When a prototypical heptad sequence (often denoted as *abcdefg*) is repeated, the resulting extended amphipathic helix undergoes self-assembly into rope-like coiled-coil supramolecular structures [48]. In order to engineer coiled-coil quaternary motifs, it is important to consider that the hydrophobic residues in the α-helix should be spaced apart by three or four hydrophilic residues. Moreover, positions *a* and *d* should be occupied by hydrophobic residues, while positions *e* and *g* are often occupied by charged residues (Figure 2) [47]. Hydrophobic interactions between residues *a* and *d*, and electrostatic interactions involving residues *e* and *g*, contribute to the stability of coiled-coil quaternary structures. Expansion of the hydrophobic core to include residues *e* and *g*, combined with restriction of ionizable residues to positions *b*, *c* and *f*, promotes oligomerization and increases stability [49,50]. Coiled-coil assemblies could implicate more than two helices, which could be oriented parallel and/or antiparallel, and the assemblies could be homo- or hetero-oligomeric.

One of the first coiled-coils used in biomedical applications was the self-assembling fiber system, which involves a peptide building block containing four successive heptads, (KIppLKp)_2_(EIppLEp)_2_, where *p* stands for polar residues [51]. The hydrophobic residues at positions *a* and *d* promote self-recognition, whereas the charged residues at positions *e* and *g* allow the formation of staggered assemblies. Such a design at the sequence level leads to a variety of different resulting fibril morphologies (i.e., length and thickness), based on the complementary principle of generating blunt or sticky ends, well established for DNA assembly. Moreover, kinks and branches can be introduced in the fibrils by rearranging the heptad units and/or by introducing branched or dendritic linker [51,52]. The thickness and length of the resulting assemblies can also be controlled by changing the residues at the position *c*, *b* and *f* [53]. The fibril morphology of a nanotube composed of a 28-residue peptide (CC-Hex-T) has been modulated by building blunt-ended α-helix barrels and by exploring a wide range of coiled-coil oligomers, including pentamers, hexamers and heptamers, without the assistance of sticky ends [54]. The evaluation of the immune responses raised by a hydrogel-forming system based on the coiled-coil domain of fibrin demonstrated that higher-order assembly modulates the immunogenicity of these particles. The triblock peptide-PEG-peptide (γKEI-PEG-γKEI) assembly raised a moderate antibody response against the scaffold, while the peptide alone (γKEI) did not [55]. A supramolecular vaccine system has been designed from the Coil29 peptide that self-assembles into elongated nanofibers composed entirely of α-helical structures [56].

### 3.2. Cross-β-Sheet and Other β-Sheet-Rich Quaternary Motifs

**Figure 3:**
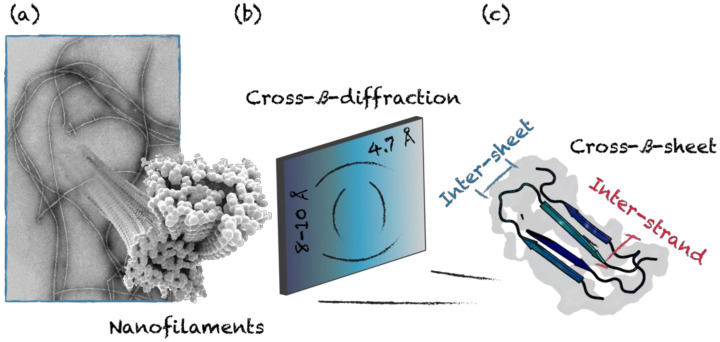
Schematic representation of cross-β-sheet suprastructure. (**a**) Representative transmission electron microscopy (TEM) image of cross-β fibrils. (**b**) X-ray diffraction pattern representing typical (**c**) inter-strand (4.7 Å) and inter-sheet (8–10 Å) distances. (PDB 2LNQ).

β-sheet-rich quaternary motifs are known for their inherent aggregative nature, which is associated with the formation of highly stable fibrils. The main sequence pattern forming β-sheets is obtained by alternating hydrophilic and hydrophobic residues. As a result, the assembled sheet has a hydrophilic and a hydrophobic face, where two sheets are brought together by excluding the surrounding water [57]. The number and the supramolecular organization of packed sheets modulate the final architecture, resulting in a diversity of mesoscopic morphologies, including tapes, ribbons and fibrils [58]. The design of short β-sheet sequences prone to self-assemble into macromolecular arrays has set the grounds for using polypeptides as building blocks for functional biomaterials [59]. Cross-β-sheet is a common self-assembling motif emerging from β-sheet rich structures, where β-strands run perpendicularly to the fibril axis [60]. This arrangement is characterized by a typical X-Ray diffraction pattern with two major diffraction peaks. An intense peak is located on the meridian at 4.7–4.8 Å, which is parallel to the fibril axis and relates to the distance between the β-stands. The second signal is located along the equatorial axis at 8–10 Å and arises from the side-chain packing within sheets (Figure 3) [61].

The cross-β-sheet architecture is associated with amyloid fibrils, which have been historically connected to numerous diseases, such as Alzheimer’s disease and systemic amyloidosis [62]. Recent biophysical and biological evidences have shown that amyloid fibrils are inert thermodynamic products of aggregation and that cytotoxicity is mainly associated with oligomers [63]. Moreover, functional amyloids have been identified in most living organisms, from bacteria to humans [64]. Owing to their high stability and mechanical properties, comparable to those of steel and spider silk, amyloids have recently emerged as a promising biomaterial [65]. Nonetheless, it has been reported that different sequences under the amyloid fold can cross-interact with endogenous proteins and promote their amyloid aggregation [66,67]. Cross-seeding by amyloid fibrils is governed by conformational supramolecular recognition and compatibility, which require a similar conformational framework. Whereas two similar dominant conformations can cross-seed each other, sufficient difference between sequences can act as a seeding barrier [68]. Cross-seeding needs to be considered when using cross-β-sheet assembling motifs in the design of nanovaccines. Numerous short synthetic peptides that self-assemble into cross-β nanostructures have been identified. For example, the Q11 peptide (Ac-QQKFQFQFEQQ-Am), which self-assembles into fibrils, has been evaluated as a vaccination scaffold (Table 1) [30]. Similarly, it was recently shown that a 10-mer peptide (SNNFGAILSS-Am) derived from the amyloidogenic peptide islet amyloid polypeptide (IAPP) conjugated to the immunogenic epitope E2EP3 from the chikungunya virus elicits a robust IgG response against E2EP3, which was dependent on self-assembly and did not require co-injection of adjuvants [69]. These two previous studies showed that the immunostimulating effect was related to the fibril morphology, suggesting that the cross-β motif can activate the innate immune system. More sophisticated supramolecular structures built on β-sheet self-assembling motifs were developed, including virus-like capsules [70]. The 24-mer β-annulus peptide (INHVGGTGGAIMAPVAVTRQLVGS) from tomato bushy stunt virus capsid was used to form synthetic spherical viral capsids. The 30–50 nm diameter β-annulus nanostructure is composed of trigonal β-sheet peptides that associate into a C3-symmetric structure, in which β-strands wrap onto a three-fold axis. The strong interactions between the sticky ends of the β-annulus structures drive aggregation, resulting in well-defined spheres [71]. This three-way component design was also extended to an artificial C3-symmetric trigonal (FKFE)_2_ peptide that forms β-sheet and self-assembles into viral-sized peptide nanospheres [72].

## 4. Supramolecular Protein Assemblies as Nanovaccine Scaffolds

Immunological properties of protein assemblies are associated with specific characteristics of the supramolecular architecture, including size and shape, charge, surface chemistry, particulate nature, multivalency and repetitive antigen display. Therefore, the ability to precisely control the morphology and properties of the proteinaceous scaffold and to functionalize them with multiple immunological components (i.e., antigens, adjuvants) with stoichiometric precision is key to modulate, potentiate and polarize the immune responses.

### 4.1. Symmetry-Based Design

**Figure 4:**
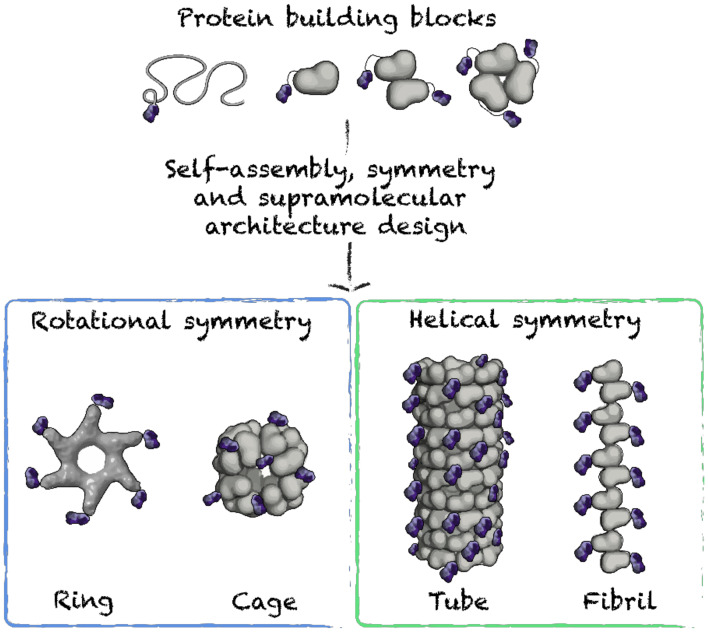
Schematic representation of supramolecular protein architectures used for nanovaccines.

In biological systems, symmetric structures are ubiquitous, as this organization leads to thermodynamically favorable supramolecular assemblies, with functions that relate to the morphology [82,83]. Nanovaccine design based on protein building blocks avidly exploits symmetry, leading to particles that mimic symmetric viral structures. Highly organized assemblies can be elaborated from engineered building blocks obeying symmetry principles (Figure 4). Symmetric nanoparticles are described by symmetry groups. Symmetry groups are defined by a set of spatial operations, i.e., rotation, translation, which allows organized interactions between the repetitive units [84,85,86]. For instance, a group having a C4 symmetry can be composed of four subunits in a cyclic arrangement, displaying the following group elements (0°, 90°, 180° and 270°). In this example, a repeated symmetry operation, i.e., 90° rotation, generates all the elements in the symmetry group. In order to engineer complex symmetric protein structures, it is important to coordinate contacting interfaces, or self-assembling motifs, in such a way that the group elements generate a full symmetry group within the entire assembly [84,85,86].

Symmetry groups can be used to understand the number and the orientation of contacts required to hold the subunits together and connect them into spatially defined objects. Accordingly, integrating oligomerization domains enabling precise contacts between de novo engineered building blocks could promote their assembly into programmed symmetric structures. Symmetry principles guiding the construction of predicted architectures based on regular polyhedra (e.g., cubic cages) are straightforward. For instance, any combination of two rotational operators is suitable for building these symmetric structures [85]. Such an approach is highly relevant to vaccination nanotechnology because it relates to the symmetry principles exploited by nature, including viruses. Comprehensive reviews have addressed the geometric principles for designing nanoparticles and have highlighted the importance of these principles in protein self-assembly [85,87]. Furthermore, a list of the different tabulations of allowed symmetry operations has recently been published, and a procedure based on symmetry definition has been implemented in Rosetta, supporting parameterization of the desired assemblies [86,88]. Interestingly, a mathematical-based package has been developed for exploiting symmetries such as icosahedral and tetrahedral, in order to precisely control the repetitive antigen display on the nanovaccine, which can generate an optimal humoral immune response [88]. For example, two covalently linked oligomers, i.e., dimeric M1 matrix protein and trimeric bromoperoxidase, assemble into a predicted orientation and lead to symmetric assemblies. The three-dimensional orientation of these two domains could be controlled by adjusting the rigidity of the helical linker, resulting in 12-subunit tetrahedral cages [86,88].

### 4.2. Nanorings, Polyhedral Cages and Nanoparticles

**Figure 5:**
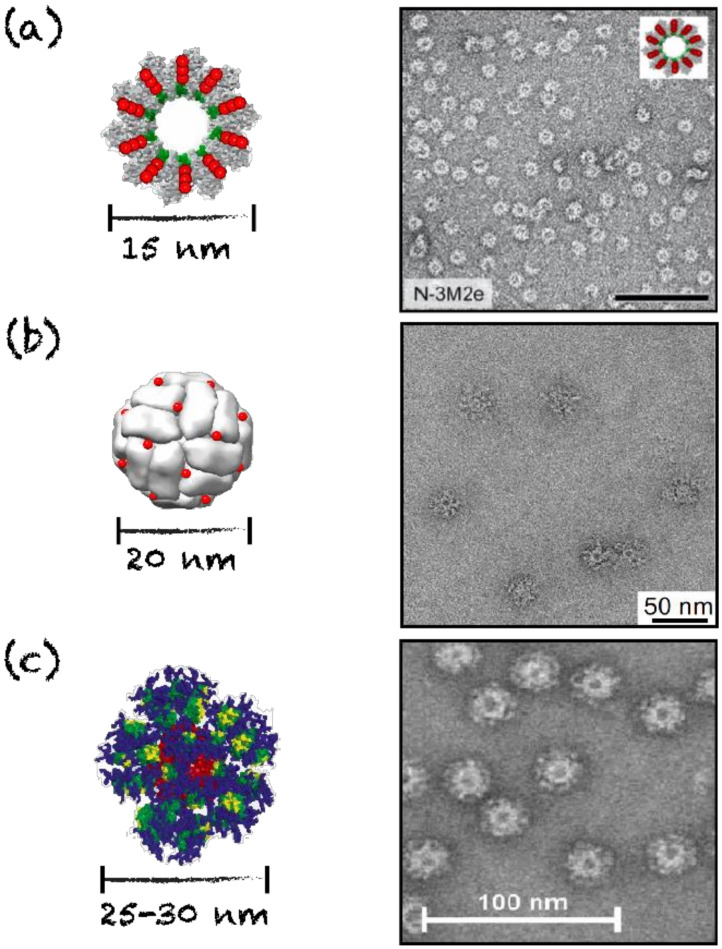
Schematic representations and TEM images of rings and cages evaluated as nanovaccines. (**a**) N-3M2e nanorings, scale bar = 100 nm. Reprinted from [73], copyright 2014, with permission from American Society for Microbiology. (**b**) D_123_-Ferritin octahedral cages. Reprinted from [76], copyright 2015, with permission from Elsevier. (**c**) eOD-GT6 60-mer lumazine synthase icosahedral cages. Reprinted from [77], copyright 2013, with permission from AAAS.

Symmetric assemblies mimicking viral capsid structures include nanorings, polyhedral cages and nanoparticles. Polyhedral nanoparticles and cages can adopt tetrahedral, cubic, octahedral, dodecahedral and icosahedral symmetries. Such assemblies, apart from mimicking natural pathogens, present additional advantages for vaccine applications, including a small size, which enables localization to the lymph nodes and efficient uptake by APCs [6]. Protein nanorings are usually unilayered discs composed of 7 to 20 protein subunits and have diameters ranging from 8 to 20 nm [73,89,90,91]. Nanorings are often the building blocks for larger protein assemblies, such as nanotubes. Rings have similar supramolecular organization to those of helical viral capsids [89,90,92]. Such engineered assemblies presenting antigens on one face of the particle have shown an enhanced antibody response, potentially attributed to immune surface receptor clustering and activation, which constitutes a key aspect for the design of nanovaccines [6,93]. For instance, nanorings composed of 10 to 11 monomers of the nucleoprotein (N) of the human respiratory syncytial virus linked to the ectodomain of the matrix 2 protein (M2e) of the influenza virus were evaluated as a vaccine against the influenza viruses (Figure 5). Oligomerization of the N-protein building blocks arises from weak interactions, such as Van der Waals contacts and polar interactions, and is stabilized by ionic interactions between α-helices. This results in 15 nm diameter monolayered rings with a helical symmetry and a central cavity [73,94,95,96]. This suprastructure is comparable to that of other helical viral capsids, such as the tobacco mosaic virus capsid and the Ebola virus nucleocapsid [90,97,98]. Upon immunization in mice, this platform has the ability to induce a specific antibody response against the grafted M2e epitope, resulting in protective immunity against influenza [73].

Similarly, nanovaccines can be engineered from the self-assembly of seven copies of IMX313, a coiled-coil heptamerizing domain of the complement C4 binding protein (C4bp). The resulting spider-like nanorings, with a diameter of 10 nm, are stabilized by hydrophobic interactions between the alpha chain of each subunit and by disulfide bonds involving the C-terminal domain [74,93,99,100,101]. Immunogenic epitopes of the malaria parasite have been conjugated to this heptameric scaffold, generating a vaccination platform inducing a high antibody response (Table 1) [74]. A *Plasmodium falciparum* malaria vaccine candidate based on Pfs25-IMX313 and with saponin-based adjuvant (Matrix-M1) is currently undergoing phase one clinical trials to assess safety and immunogenicity (NCT04130282). However, usage of nanorings as antigen delivery scaffold is limited by the fact that self-recognition can be difficult to control precisely and that the repetitive antigen display on these particles is limited by their small size and low oligomerization state [73,100,101].

Vaccination against viral infections has exploited synthetic polyhedral cages and nanoparticles as scaffolds, owing to their size, symmetry and ordered quaternary architecture, which resemble those of viral capsids [102,103,104,105]. This supramolecular organization allows repetitive antigen display in a way that enables efficient immune cell activation through surface receptor clustering [103,104]. Of regular polyhedra, the icosahedron is often used due to its close similarity to the shape and structure of numerous viral capsids. These structures are assembled from coiled-coil motifs, which are derived from proteins (e.g., ferritin and lumazine synthase) or from de novo synthetic peptides [102,105,106]. For instance, uniform icosahedral nanoparticles were assembled from a synthetic peptide composed of (i) a pentameric coiled-coil domain from the cartilage oligomerization matrix protein, (ii) a short Gly–Gly linker and (ii) a trimeric de novo designed coiled-coil domain [75,102]. These chimeric peptides first oligomerize into 15-mer assemblies, which is the least common multiple of the two domains oligomerization states. Then, three or four of these oligomers self-assemble into dodecahedral and icosahedral nanoparticles, respectively composed of 45 and 60 subunits [75,102]. Immunogenic epitopes can be conjugated to these synthetic peptides, which can still assemble into icosahedral nanoparticles displaying the selected epitope on their surface. This nanoplatform decorated with tandem repeats of a B-cell immunodominant epitope of the malaria parasite induced a strong immune response and led to protection against experimental infection of *Plasmodium* sporozoites in mice (Table 1) [75].

Lumazine synthase-based cages are composed of 60 subunits that self-assemble into 20 to 25 nm icosahedral nanoparticles, highly resembling viral capsids (Figure 5) [78,105]. An epitope of the gp120 glycoprotein from human immunodeficiency 1 virus was conjugated to these nanoparticles and induced specific antibody response in mice [77,78]. Moreover, nanoparticles based on ferritin that assemble into 20 nm hollow octahedral nanoparticles of 24 subunits were evaluated as antigen delivery (Table 1) [76,106,107]. These particles are composed of twelve dimerization interfaces, eight trimerization interfaces and six tetramerization interfaces, most probably divided in two even units of 12 protein subunits [102,107]. An octahedral cage of ferritin conjugated to an epitope of the gp350 glycoprotein from the Epstein–Barr virus induced a strong induction of neutralizing antibodies and a protective immunity against viral infection in a mouse model (Figure 5b) [76]. The hollow nature of cages also offers the possibility of encapsulating polynucleotides, mimicking virus natural composition. For example, the E2 subunit of the pyruvate dehydrogenase complex can self-assemble in a 30 nm dodecahedral hollow cage composed of 60 subunits, following the symmetry principles previously described [79,108]. Not only can these nanoparticles can be functionalized on their surface with viral epitopes through chemical crosslinking, but they can simultaneously be functionalized chemically on internal cysteine residues with the TLR9 agonist CpG 1826. This divalent platform induces a specific cellular response and delays tumor growth in mice [79,108]. These nanocages are smaller than the majority of virus icosahedral capsids, such as adenoviruses and herpesviruses, whose capsid measures approximately 100 nm in diameter [109,110]. Nonetheless, cage nanoparticles are structurally similar to many plant viruses, such as the cowpea mosaic virus, and their size range (20 to 35 nm) is optimal for direct diffusion into the lymph vessels and uptake by APCs [6,89].

### 4.3. Nanofilaments and Nanotubes

**Figure 6:**
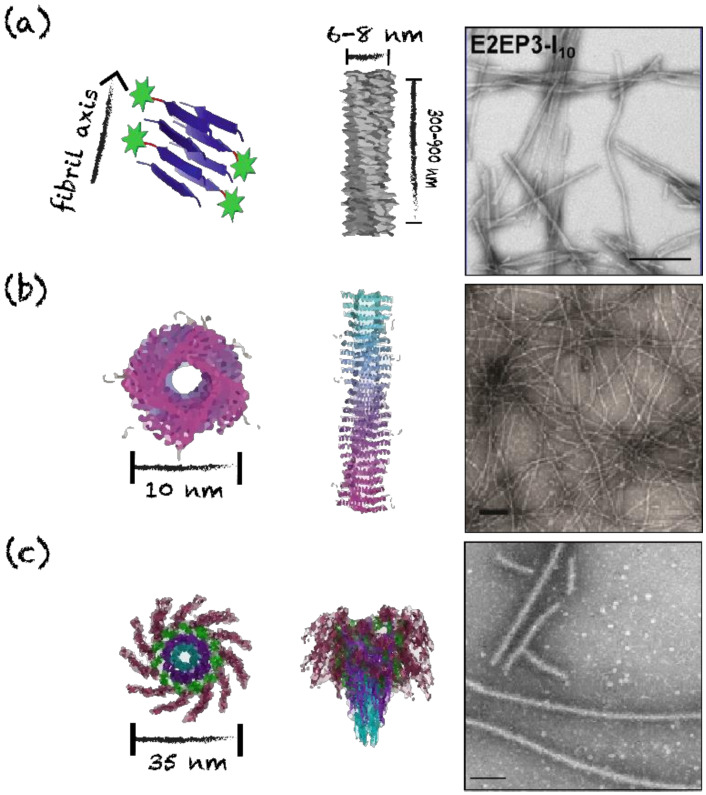
Schematic representations and TEM images of proteinaceous filaments and nanotubes. (**a**) E2EP3-I_10_ nanofibrils, scale bar = 200 nm. Adapted from [69]. (**b**) PEP-C/PAD-C Coil29 nanofibers, scale bar = 100 nm. Reprinted from [56], copyright 2017, with permission from American Chemistry Society. (**c**) hFliC flagellin nanofilaments, scale bar = 500 nm. Reprinted from [81], copyright 2015, with permission from Springer Nature.

Linear, long and unbranched assemblies have been evaluated as nanovaccine scaffolds, including nanofilaments (or fibrils) and nanotubes. In contrast to fibrils, nanotubes are characterized by a hollow center cavity. The structure of these assemblies geometrically resembles the conformation of numerous pathogen-associated suprastructures, such as bacterial flagella and pili, helical viral capsids, and filaments from enterobacterial biofilms [6,89,111,112]. These elongated assemblies allow the stabilization and the repetitive display of antigens that can potentially activate T cell-independent immune responses, if the nanoscaffold is longer than 500 nm [6,80,81]. Furthermore, the cross-β quaternary conformation has shown some intrinsic adjuvating properties [30,113,114]. These fibrillar platforms, with a length that can reach over 1 μm, have a diameter between 5 and 15 nm and are characterized by high polymorphisms in terms of length and morphology, i.e., twisted filaments vs. flat ribbons [30,32,69,115]. As described above, the Q11 synthetic peptide self-assembles into micron-length cross-β unbranched fibrils with a diameter of approximately 10 nm [30]. Mice immunization with assembled chimeric peptides composed of Q11 and OVA_323−339_, a T- and B-cell epitope of ovalbumin, elicited high levels of IgG1, IgG2a and IgG3 antibodies against the OVA epitope. The level of antibodies induced by these assemblies was comparable to the one reached by the OVA-decorated fibrils co-administrated with the complete Freund’s adjuvant, showing strong immunogenicity [30]. Different antigenic determinants have been conjugated to this scaffold, including glycopeptides for cancer therapy, J14 epitope from the M protein of group A streptococcus and the malaria-based NANP epitope from the circumsporozoite protein [80,114,116]. Mice immunization with Q11 fibrils, decorated with three tandem repeats of NANP sequence from *Plasmodium falciparum*, induces a potent anti-NANP IgG response and confers protection against infection with *Plasmodium* sporozoites in mice (Table 1) [80]. Another example is the chimeric E2EP3-I10 peptide, which was used as a vaccine platform against the Chikungunya virus (Figure 6). This peptide spontaneously self-assembles into 300 to 900 nm long poorly twisted fibrils with a diameter of 6 to 8 nm [69].

Considering that numerous viral helical capsids and bacterial flagella and pili have a nanotube morphology, coiled-coil based nanotubes constitute promising vaccination platforms [89,91,112,117]. For instance, well-defined nanotubes with diameter of 6 to 10 nm can be obtained from the self-assembly of Coil29 (Figure 6). Nanotubes formed from the co-assembly of Coil29 conjugated with a cancer B-cell epitope from the epidermal growth factor receptor class III variant (EGFRvIII), and Coil29 linked to the universal CD4+ T-cell epitope PADRE, are readily internalized by APCs and induce robust antibody production, CD4+ T-cell and CD8+ T-cell responses in mice [56]. Moreover, the TLR5 agonist flagellin can assemble into coiled-coil-based nanotubes, mimicking the morphology of bacterial flagella, and these filaments have been evaluated as subunit vaccine scaffolds [81,118,119,120]. For instance, the D3 solvent-exposed domain of the flagellin from Salmonella, FliC, was replaced by the DENV2 epitope of the Dengue virus envelope protein. This chimeric DENV2-FliC protein, which conserved the D0 and D1 self-assembling domains, forms long filaments with a diameter of 35 nm and a central hollow cavity (Figure 6). The resulting nanotubes induce T-dependent responses in C57BL/6 mice, as well as T-cell-independent specific antibody responses in the 6.5-TCR murine model lacking T-cell receptors (TCRs). Accordingly, flagellin-based nanotubes may represent a promising avenue for the vaccination of immunocompromised patients.

### 4.4. Antigen Functionalization on Protein Supramolecular Structure

**Figure 7:**
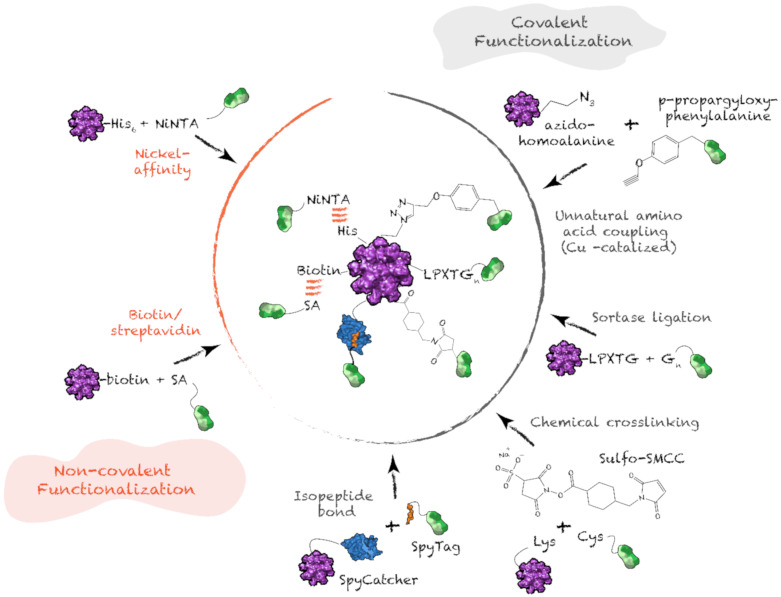
Covalent and non-covalent post-assembly functionalization. SA: streptavidin; G_n_: polyglycine motif.

Protein nanostructures designed for vaccination are hybrid supramolecules composed of at least three domains: (i) self-assembling moiety or scaffold; (ii) antigenic determinant(s); (iii) linker(s); and, occasionally, (iv) a molecule(s) with adjuvant properties. Most commonly, the epitope is covalently linked to the building block unit prior to self-assembly. In such cases, a flexible sequence, most frequently enriched in Gly and Ser residues, is incorporated between the two moieties. This linker allows independent folding and/or appropriate self-assembly. For small proteins, i.e., below 40 to 50 residues, the carrier-linker-epitope hybrid sequence can be obtained by solid phase peptide synthesis [69,80,121]. Most commonly, chimeric building blocks are obtained through traditional strategies for recombinant protein expression. [73,76,81]. Although this preassembly functionalization strategy usually allows efficient folding and self-assembly, the chimeric sequence encompassing the scaffold and the antigen can compromise self-assembly and/or cause misfolding of the self-assembling unit or antigen(s) [121,122]. These issues can be addressed with post-assembly orthogonal functionalization (Figure 7). These strategies not only support independent folding and self-assembly, but also enable efficient substitution of a diversity of epitopes on a carrier nanoparticle, addressing the challenge of antigenic drift [123,124].

Non-covalent functionalization methods can take advantage of the moderate affinity between a poly-His sequence and nickel-coordinated nitrilotriacetic acid (Ni-NTA), or the strong affinity between streptavidin and biotin. NTA can be chemically conjugated to a lysine side-chain of the epitopes and coordinated with nickel ions, allowing non-covalent bonding to preassembled protein nanostructures bearing accessible poly-His [125,126]. Since the affinity between poly-His sequence and Ni-NTA is moderate, this interaction is reversible, and, at a pH below the pka of the imidazolium side-chain, the interaction becomes unstable [127]. In contrast, interaction between streptavidin, which can be linked to the self-assembling building block via genetic fusion, and the biotinylated epitope leads to highly stable nanoparticles [128,129].

Post-assembly covalent functionalization of protein nanoparticles with antigenic determinants and/or adjuvants confers high stability to nanovaccines. Primary amine, i.e., N-terminus and Lys side-chain, and thiol, i.e., Cys side-chain, are often harnessed for chemical crosslinking. Chemical ligation allows functionalization with non-protein molecules presenting adjuvant properties, such as polysaccharides, lipids and polynucleotides. For example, in the CpG-gp-E2 vaccine, the preassembled dodecahedral cage was functionalized on surface-exposed Lys with succinimide-conjugated glycoproteins, while the encapsulated CpG oligonucleotide was bound to internal Cys using maleimide crosslinking [79,108]. Trimeric coiled-coil peptides have also been chemically conjugated with haptens prior to self-assembly, to generate anti-nicotine vaccines [130]. However, these strategies often suffer from poor orthogonality and specificity. Functionalization on Cys can interfere with the formation of preexisting disulfide bonds within the carrier or the antigen. For orthogonality and control over stoichiometry, incorporation of unnatural amino acids enables *click chemistry*, which is fast, selective and can be performed in aqueous solution. For example, azidohomoalanine can be introduced as a Met surrogate, while *p*-propargyloxy-phenylalanine can replace Phe or Tyr [131,132]. Azido and alkyne undergo copper-catalyzed cycloaddition, leading to covalent linkage of the two moieties [133]. Although this functionalization strategy requires minimal changes in the primary sequence, it is not widely used, because of the challenges associated with the incorporation of unnatural amino acids in recombinant systems.

Accordingly, conjugation strategies involving natural amino acid sequences are needed in most cases. Specificity of functionalization can be achieved by using sortase, a bacterial enzyme that catalyzes transpeptidation of a protein containing a terminal LPXTG motif with a protein bearing a terminal polyglycine motif [134,135]. This strategy has been used to covalently link N-terminally LPETGG-labeled nanoparticle scaffolds, such as virus-like particles and E2 dodecahedral cages, with terminally GGGS-labeled proteins [134,135,136]. Recently, the SpyTag/SpyCatcher technology has been used for irreversible conjugation of antigens on preassembled carrier nanoparticles [137,138,139]. This system is based on a 13-residue sequence (SpyTag) that forms an intermolecular isopeptide bond with a 116-residue protein (SpyCatcher), with both sequences being extracted from the collagen adhesin domain of the fibronectin binding protein of *Streptococcus* [139,140,141]. These functionalization methods are efficient, orthogonal and confer high stability to the nanoparticles. Recent development of other catcher/tag technologies has enabled precise control over functionalization with multiple epitopes [74,142,143,144]. For example, precise antigen titration of the Pfs25 and Pfs28 epitopes from the malaria parasite on IMX313-based spider-like nanorings was achieved by using the orthogonal Spy and Snoop catcher/tag conjugation strategies [76]. Considering that functionalization of nanoparticles with multiple different epitopes is often needed to induce protective immunity against pathogens, such orthogonal labeling strategies are important for nanovaccine development [74,132].

## 5. Tuning the Immune Response with Protein Nanostructures

Proteinaceous supramolecular structures constitute promising scaffolds for subunit vaccines, as these ordered assemblies can simultaneously act as an antigen delivery system and immunostimulants. Advancements in the understanding of the mechanisms of action of subunit nanovaccines have highlighted the importance of the activation of innate immunity through simultaneous pathways to trigger a robust adaptive immune response and promote long-lasting protective immunity. In this context, it is important to understand how the physicochemical and structural properties of nano-sized objects modulate and potentiate immune responses. In this section, we briefly present the immunological mechanisms involved in vaccination and emphasize the key characteristics of protein nanostructures, i.e., size, mesoscopic morphology, charge, hydrophobicity and intrinsic adjuvanticity.

### 5.1. Induction of an Adaptive Immune Response with Protein Nanostructures

**Figure 8:**
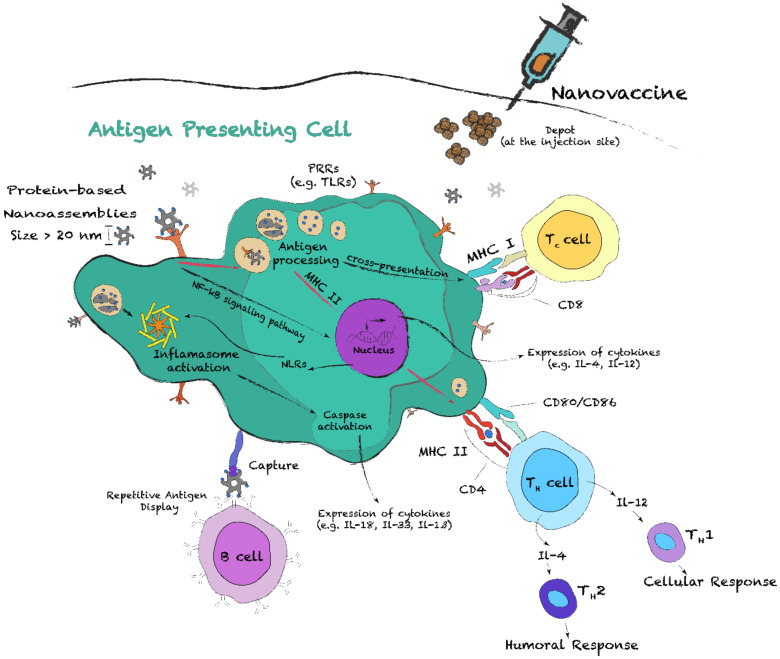
Schematic representation of the contributions of antigen presenting cells in the adaptive immune response following vaccination with protein nanoassemblies. The nanoparticles can be recognized by pattern-recognition receptors (PRRs), such as Toll-like receptors (TLRs). PRRs trigger intracellular NF-κB signaling pathways, resulting in the expression of pro-inflammatory cytokines. After internalization and processing of the nanoparticles, APCs can present antigens to T helper (Th), CD4+, cells via the major histocompatibility complex (MHC) II and, by cross-presentation, to T cytotoxic (Tc) cells, CD8+, via MHC I. Cytokine stimulation of activated Th cells promotes their differentiation into Th1 and Th2 subgroups, which respectively promotes cellular and humoral immune responses.

The induction of an effective protective immunity by subunit nanovaccines includes a series of key steps involving the innate and adaptive immune systems. One of these critical events consists of the phagocytose and processing of the nanoparticles by APCs, which include dendritic cells (DCs), macrophages and B cells (Figure 8) [6]. Particularly, DCs and, to a certain extent, macrophages are both highly specialized at this level and are crucial for the T-cell and B-cell responses [145,146]. Before antigen encounter, DCs, which are mostly located in peripheral tissues, are at an immature state, constantly sampling their environment by macropinocytosis, phagocytosis and/or receptor-mediated endocytosis. During this process, they mostly encounter apoptotic cells and self-antigens, keeping cells under tolerance steady state [145]. When an infection occurs, or upon vaccination, APCs uptake the pathogens, or the nanoparticles. Pathogen identification can occur through PAMPs recognition by PRRs, such as TLRs, scavenger receptors (SRs) and nucleotide-binding oligomerization domain (NOD)-like receptors (NLRs) [147,148,149]. Upon activation of these conserved innate immune receptors, extensive changes in metabolism and gene expression occur, such as the activation of the NF-kB signaling pathway, which results in cytokine secretion and leads to DC activation and maturation [149]. This constitutes another critical event for linking innate and adaptative immunity. Activated DCs will (i) migrate toward secondary lymphoid organs, (ii) upregulate the expression of major histocompatibility complex (MHC) molecules and co-stimulatory molecules (CD80, CD86), and (iii) secrete various cytokines (Figure 8) [149,150]. Together, these modifications prime T cells and promote their differentiation into effector subtypes.

Activation of T cells occurs following the interaction of the T-cell receptors (TCRs) with antigens bound to MHC II and/or MHC I molecules at the surface of APCs. All nucleated cells express MHC I, while only APCs express both MHC II and MHC I (Figure 8). Antigens displayed on MHC I are considered endogenous, as they result from proteolytic degradation of intracellular proteins. APCs, however, can display exogenous antigens on MHC II and MHC I, by cross-presentation (CP). Among APCs, DCs are known for their unique capacity to cross-present antigens [151]. Antigen presentation on MHC II results in activation of CD4+ T helper (Th) cells and differentiation into effector subsets, while presentation on MHC class I activates CD8+ T cytotoxic (Tc) cells. Subsets of Th cells include Th1, Th2, Th17 and Treg [152,153]. On the one hand, Th1 cells secrete IFN-γ and IL-2 and are associated with cell-mediated immune response, i.e., against intracellular pathogens. On the other hand, Th2 cells secrete IL-4, IL-5, IL-9 and IL-13 and are associated with the induction of a humoral immune response [153]. The balance between Th1 and Th2 immune responses is key for vaccination, as polarization toward Th1 usually generates more effective antiviral response [154]. Imbalance in Th1/Th2 polarization by nanovaccines can be overcome by controlling the nanoparticle characteristics and/or by incorporating adjuvant molecules in vaccine preparations [155]. Finally, B cells, whose differentiation results in memory B cells and antibody-secreting plasma cells, can sense antigens through direct interaction with membrane bound IgM or IgD [6,153]. Crosstalk between Th2 cells and B cells is critical to generate high-affinity antibodies and isotype switching [156].

### 5.2. Modulating Immune Responses with Tailored Protein Nanostructures

Understanding how the structural and physicochemical properties of protein supramolecular structures modulate the potency and the polarization of the immune response is critical for bottom-up design. Characteristics such as size, repetitive antigen display, charge, surface hydrophobicity and PRR activation, dictate the immunogenicity of the nanoparticles. Accordingly, guiding these parameters allows a unique control over key aspects of the immune responses.

Particle size and antigen display: APC uptake and processing pathways are size-dependent, but also depend on the type of APCs [157]. For soluble subunit antigens with diameters below 10 nm, uptake is generally low compared to nanoparticles with diameters over 20 nm [6,158,159]. When the nanoparticle diameter is comprised within the nanometer range (20–1000 nm), mechanisms of cellular uptake and antigen processing by DCs and MCs differ from soluble antigens. For instance, the uptake of nanoparticles with diameters ranging from 20 to 200 nm by DCs via macropinocytosis is more important than MCs’ uptake. In contrast, large particles (1–3 µm) are mainly uptaken by MCs via phagocytosis and receptor-mediated endocytosis [160,161]. These microparticles can also be captured by subcapsular sinus MCs and some DCs, allowing transportation to B cells in lymph nodes [6,162]. Several studies have reported that nanoparticles with a diameter below 150 nm enter APCs through clathrin, or caveolae, endocytosis, a pathway also used by many pathogens, including the influenza and the respiratory syncytial viruses [154,163,164]. In contrast to microparticles, nanosized assemblies (Table 1) lead to preferential uptake by DCs, which in turn stimulates cytotoxic CD8+ cell priming and CP of the antigens [165,166]. Nonetheless, as the mechanism of CP remains poorly understood, it is not clear if this effect is associated with endosomal escape, or with differential trafficking of the endosomes [151]. The size of the protein assemblies, i.e., low nano vs. micro scale, appears to have some influence on the polarization of the immune response [167]. Interestingly, small nanoassemblies (<200 nm) with a dense organized antigen display can reach the lymph nodes in a native form and directly activate B cells in a T-cell independent manner, by crosslinking and clustering cell-surface immunoglobulins, such as membrane IgD [6,168]. This type of response was also observed for larger nanoassemblies, such as the previously described hFliC filaments [81]. Subsequent signaling pathways trigger activation of B cells, which can secrete specific immunoglobulins, such as IgM [169]. Pentameric IgM antibodies efficiently bind repetitive antigen displays, leading to the recruitment of the complement component 1q (C1q). Activation of the complement pathway enhances uptake by APCs through the formation of immune complexes (ICs) and/or opsonization [6]. ICs can be captured by follicular DCs (Figure 8) in B-cell follicles of the lymph nodes, playing a critical role in B-cell maturation [170,171].

Moreover, due to their high surface-to-volume ratio, nanoparticles present an increased antigen exposure to the biological environment, resulting in an enhanced antigenicity and immunogenicity [75,172]. Indeed, the number of antigenic moieties per nanoparticle is also a parameter that needs to be considered in nanovaccine design. For soluble antigenic determinants, with the exception of multivalent antigens, the number of antigens in the endosomes of APCs equals the number of particles internalized [173]. In sharp contrast, higher concentrations of antigens can be achieved by decorating nanoparticles with multiple copies of the antigen [174]. For example, the eOD-GT6 lumazine synthase icosahedral cages (Figure 5c), which structurally resemble viral capsids, present 60 copies of the gp120 HIV-1 epitope on their surface, enhancing the antibody response [77]. Similarly, other protein nanostructures, which are presented in Table 1, have been constantly reported to induce a much higher immune response against the grafted epitope in comparison to the soluble epitope, even in presence of Alum-based adjuvant [69]. Moreover, nanoclusters of HA trimers and M2e tetramers were shown to enhance TNF-α expression and to increase IgG titers in comparison to their soluble forms [175,176]. The size and the repetitive epitope display of such nanostructures mimic most viral surfaces, where a limited number of proteins are displayed in a highly organized manner [177,178]. Such ordered architecture is recognized by components of the innate immune system, which allows the establishment of a robust immune response. Moreover, as size and geometry play such an important role in antigen delivery and immune responses, one would hypothesize that the scaffold architecture needs to be well controlled and the vaccine preparations present a high homogeneity. Low polymorphism should better mimic natural pathogens, which are uniform in size, geometry and morphology, and likely represent an optimal scaffold for vaccination [179,180]. While small particles efficiently diffuse into the lymph nodes and are readily uptaken by APCs, large platforms enable efficient antigen capture, transportation to B cells and potential activation of T-cell-independent responses. Accordingly, the use of hybrid vaccine formulations, composed of particles with different size ranges could ultimately enhance immune stimulation by potentially increasing the variety of mechanisms involved in antigen detection and processing, which could be advantageous for engineering vaccine scaffolds [181].

Surface properties: Surface properties of protein nanostructures, such as charge and hydrophobicity, also modulate the immune responses. Positively charged nanoparticles generally trigger a robust inflammatory response compared to anionic and neutral particles [182]. For instance, studies on Q11 cross-β fibrils have shown that the addition of positive charges enhances APC uptake and MHC class II presentation of peptides to T cells, which results in an increase of immunogenicity. In contrast, the presence of negative charges on the same self-assembling β-peptides led to a sharp decrease of the immunogenicity [183]. As observed for cell-penetrating peptides, electrostatic interactions with the negatively charged outer leaflet of the plasma membrane are known to enhance cellular uptake [184,185]. Surface-exposed hydrophobic clusters are considered damage-associated molecular patterns (DAMPs), stimulating APC uptake and local inflammation [186]. For example, by using a small library of gold nanoparticles functionalized with chemical identities with increasing hydrophobicity, it was reported that surface hydrophobicity increases inflammation, as measured with expression of the pro-inflammatory cytokine TNFα. However, the highly hydrophobic gold nanoparticles did not lead to a strong immune response, most likely related to poor biodistribution [187]. Hydrophobicity also plays a key role for the activation of the innate immune system [186]. For instance, TLRs and SRs are promiscuous receptors that recognize a broad range of hydrophobic motifs, such as lipidated peptides and lipidated saccharides [186,188]. Moreover, the hydrophobic cross-β motif can be associated with pro-inflammatory effects. Indeed, TLR2 and inflammasome activation has been reported for amyloids assembled from Aβ peptide and IAPP [189,190,191]. The activation of the inflammasome, which is mediated by a subgroup of NLRs, leads to caspase-1 activation and causes the cleavage of pro-IL-1β and pro-IL-18 (Figure 8) [192]. This leads to the recruitment of immune cells into the tissue.

TLR4 activation can be triggered not only by hydrophobic lipopolysaccharides (LPS), but also by bacterial fimbriae subunits and the fusion protein of respiratory syncytial virus [186,193]. SRs, which are associated with the NF-κB signaling pathway, are also important in bacterial detection and their clearance [194]. SR activity is mediated by the interaction with hydrophobic LPS and lipoteichoic acid, which are respectively components of the outer membrane of Gram-negative bacteria and of the cell wall of Gram-positive bacteria [195]. Analogously, exposing hydrophobic clusters on protein nanostructures can trigger innate immune receptors and confer intrinsic adjuvant properties to the assemblies [174,186,187]. Surface charge and hydrophobicity are also key parameters modulating adsorption by the host organism, as well as the biodistribution of the nanovaccine. Upon injection, charged and hydrophobic nanoparticles are rapidly coated with plasma proteins [196]. Denaturation of these host proteins following adsorption and the subsequent exposure of hydrophobic clusters can lead to local inflammation, as observed for albumin-coated nanoparticles [197]. Immunoglobulins and complement components also bind non-specifically and enhance phagocytosis by opsonization, which in turn affects biodistribution [198]. However, it remains difficult to fully appreciate the contributions of each parameter, considering the low specificity and large array of possible interactions provided by hydrophobicity and surface charges.

Functionalization with adjuvants: Adjuvants are organic or inorganic substances that enhance the antigen-specific immune response in vaccines [199]. Their effects on the immune system include the formation of a depot for the slow release of antigens and the stimulation of APCs for enhanced phagocytosis and maturation [200]. In turn, this can modulate and polarize immune responses toward Th1 or Th2, promoting cell-mediated or humoral immunity, respectively (Figure 8). For example, aluminum salts mainly improve the humoral immune response, while CpG oligonucleotide enhances cell-mediated immune responses [199,201]. However, the usage of many adjuvants, including TLR agonists, in vaccination is hampered by their rapid clearance, low stability, potential off-target inflammation and high toxicity, and by the fact that they need to be transported with the antigen in the same APC [202]. Accordingly, molecular self-assembly allows the incorporation of adjuvant moieties with the antigens on the same protein nanostructures. The design of such multivalent nanoparticles will allow potentiation and polarization of the immune response.

Immuno-stimulating molecules, such as TLR ligands, are of critical importance for DC maturation and efficient priming of T cells. TLR engagement can modulate DC metabolism, increase macropinocytosis, membrane transport and fusion events, and enhance MHC I and MHC II presentation [203,204,205]. TLR activation can also lead to internalization of the TLR-ligand complex. This induces preferential loading and presentation on MHCs of antigens that were co-localizing with TLR ligands in the phagosomes [147]. In fact, administration of soluble adjuvants and antigens separately leads to poor immunogenicity [206]. Thus, supramolecular protein assemblies displaying both the antigens and the TLR ligands on the same particles provide optimal antigen-specific APC activation. Furthermore, the inherent inflammation and potential side effects of certain adjuvants may be limited by covalent conjugation and/or co-packaging the TLR ligand with the antigen on the same nanoparticles [6]. Accordingly, only the cells that have encountered the antigen will be activated by the TLR ligand, thereby reducing the necessary dose of both antigen and the adjuvant [6,207,208]. For example, CpG-gp-E2 dodecahedral cages incorporating the TLR-9 ligand CpG (Table 1) were shown to induce an enhanced cell-mediated response compared to gp-E2 nanoparticles co-administered with soluble CpG [79]. Furthermore, modular co-assembly of ferritin-HA and ferritin-flagellin, a known TLR5 ligand, revealed robust protection against influenza viral challenge without the use of other adjuvants [209]. Compared to ferritin-HA nanoparticles only, incorporation of flagellin enhanced the humoral and the cell-mediated immune responses. Thus, conjugation, or co-assembly, of TLR ligands with antigens on the same protein nanoparticles leads to increased DC activity and to a fine modulation of the immune responses.

## 6. Concluding Remarks

The continuous emergence of novel pathogens and the fact that no vaccines exist for numerous ongoing infectious diseases reinforce the importance of developing safe, scalable, efficient and affordable vaccination technologies. Subunit vaccines represent an alternative strategy to vaccine formulations based on live-attenuated and inactivated whole pathogens, although they suffer from weak immunogenicity and necessitate the addition of adjuvants. The mechanisms of action of numerous adjuvants are generally non-specific and still poorly characterized, and these molecules are often associated with side effects. By combining the properties of an antigen delivery system with those of an immunostimulant, protein supramolecular assemblies offer a promising alternative to conventional vaccines. Antigenic determinants repetitively displayed on the nanoparticle exhibit distinct immunological properties compared to their soluble counterpart. By guiding the self-recognition process at the molecular level to control the size, morphology, surface chemistry and symmetry of the nanoassemblies, we can envision the modulation of key aspects of the immune responses, including uptake by APCs, biodistribution, antigen CP and activation of PRRs. Nonetheless, future works are still needed to better elucidate how the conformation and the supramolecular architecture of protein-based nanoparticles influence their interactions with components of the immune system.

## Figures and Tables

**Table 1:** Structural and immunological properties of self-assembled protein supramolecular structures evaluated as vaccine candidates.

Candidate	Scaffold	Assembling Motifs	Morphology	Diameter (nm)	Target	Epitope	Linker	Route	Immune Response	Ref.
**N-3M2e**	Nucleo-protein N	Coiled-like	10 to 11-mer rings	15	Influenza A	(M2e)_3_	EL	IN *	↑ IgG (IgG1 and IgG2a) and IgA titers	[73]
**Pfs25:IMX: Pfs28**	IMX313	Coiled-coil	Heptameric spider-like rings	10	Malaria *P. falciparum*	Pfs25 * Pfs28 *	Snoop and Spy Tag/Catcher	IM *	↑ IgG titers	[74]
**P4c-Mal**	SAP	Coiled-coil	Icosahedral nanoparticle	25	Malaria *P. berghei*	(DPPPPNPN)_2_D	GSG	IP *	↑ IgG (IgG1, IgG2a, IgG2) titers 100% PI *, Th1/Th2 response	[75]
**D_123_-ferritin**	Ferritin	Coiled-coil	Octahedral cage	20	EBV *	gp350	(SG_3_)_2_	IM *	↑ IgG titers, PI	[76]
**eOD-GT6** **eOD-GT8**	LS *	Coiled-coil	Icosahedral cage	25–30	HIV-1	eOD * of gp120	GS linker	IP *	↑ IgG and IgM titers	[77,78]
**CpG-gp-E2**	E2	Coiled-coil	Dodecahedral cage	30	Cancer melanoma	gp100 CpG	Chemical crosslinkers	SC *	Activation of Ag-specific * TC cells, tumor growth delay	[79]
**(NANP)_3_-Q11**	Q11	Cross-β-sheet	Unbranched fibrils	15	Malaria *P. falciparum*	3 x NANP from CSP	SGSG	SC *	↑ IgG (T-cell-mediated)	[80]
**E2EP3-I10**	I10	Cross-β-sheet	Unbranched fibrils	6–8	Chikungunya virus	E2EP3	GGGG	SC *	↑ IgG (IgG1, IgG2a, IgG2b and IgG3)	[69]
**PEP-C/PAD-C**	Coil29	Coiled-coil	Unbranched fibers	6–10	Glyoblastoma	PEPvIII, PADRE	SGSG	IP *	Efficient internalization by APCs, ↑ IgG (IgG1, IgG2a and IgG2b) titers	[56]
**hFliC**	FliC	Coiled-coil	Filaments	35	Dengue virus	DENV2 E	(GGGS)_2_	IN * IP *	↑ IgG (IgG1), IgA titers ↑ IgG, IgM titers, TD * and TI * responses	[81]

* LS, lumazine synthase; EBV, Epstein–Barr virus; Pfs, plasmodium falciparum sporozoite; eOD-GT, engineered outer domain germline targeting; IN, intranasal; IM, intramuscular; IP, intraperitoneal; SC, subcutaneous; PI, protective immunity; nAbs, neutralizing antibodies; Ag, antigen; TD, T-cell dependent; TI, T-cell independent; ↑, increase.
